# NO up-regulates migraine-related CGRP via activation of an Akt/GSK-3β/NF-κB signaling cascade in trigeminal ganglion neurons

**DOI:** 10.18632/aging.103031

**Published:** 2020-04-10

**Authors:** Gang Yao, Yu-Hong Man, An-Ran Li, Yu Guo, Yun Dai, Ping Wang, Yi-Fa Zhou

**Affiliations:** 1 Department of Neurology, The Second Hospital of Jilin University, Changchun, Jilin, China; 2 School of Life Sciences, Northeast Normal University, Changchun, Jilin, China; 3 Laboratory of Cancer Precision Medicine, The First Hospital of Jilin University, Changchun, Jilin, China; 4 Department of Otolaryngology - Head and Neck Surgery, The First Hospital of Jilin University, Changchun, Jilin, China

**Keywords:** nitric oxide, glycogen synthase kinase 3β, NF-κB, calcitonin gene-related peptide, trigeminal ganglion neuron

## Abstract

The release of the neuropeptide CGRP from the trigeminal ganglion neurons (TGNs) plays a central role in migraine. Whereas CGRP can activate NO release from ganglionic glial cells, NO in turn enhances CGRP release. However, it remains unclear how NO promotes CGRP release. Here, we report that the NO donor SNAP triggered CGRP release from cultured primary TGNs. This event was associated with GSK-3β activation and Akt inactivation. Immunofluorescent staining revealed that GSK-3β primarily located in neurons. Furthermore, GSK-3β inhibition resulted in a marked reduction in expression of CGRP as well as other migraine-related factors, including substance P, cholecystokinin, and prostaglandin E2. Last, exposure to SNAP also activated NF-κB, while NF-κB inhibition prevented the induction of CGRP by SNAP. Interestingly, this event was blocked by GSK-3β inhibition, in association with inhibition of NF-κB/p65 expression and nuclear translocation. Together, these findings argue that NO could stimulate TGNs to release of CGRP as well as other migraine-related factors, likely by activating GSK-3β, providing a novel mechanism underlying a potential feed-forward loop between NO and CGRP in migraine. They also raise a possibility that GSK-3β might act to trigger migraine through activation of NF-κB, suggesting a link between neuroinflammation and migraine.

## INTRODUCTION

Migraine is a complex neurological disease, which represents the second largest cause of disability in the world. This chronic disorder, which is characterized by recurrent attacks of moderate-to-severe debilitating and suffering headaches, affects approximately 15% of the population worldwide [1]. Currently, selective serotonin or 5-hydroxytryptamine (5-HT_1B/1D_) receptor agonists (e.g., triptans) are the best available medicines for the treatment of migraine [2]. Because this class of agents act as acute anti-migraine medicines primarily by disrupting the communications between peripheral and central trigeminovascular neurons, they are more effective to prevent initiation of headache when given early before the development of central sensitization [2]. However, the success of these and other medicines for the prevention and abortion of migraine is largely unmet due to poor efficacy, tolerability and patient compliance [3–5]. As a consequence, migraine remains the leading cause of neurological disability and one of the top five causes of all long-term disability [6]. Migraine was long considered as a vascular disorder that was primarily caused by meningeal vasodilation [7]. However, the introduction of the trigeminovascular hypothesis, which proposed a central role of the trigeminal nerve and its vasoactive neuropeptide-containing axonal projections to the meninges and its blood vessels, has led to a fundamental paradigm shift - from the classic vascular disorder to a neural disease, in mechanistic understanding and clinical management of migraine [2]. In this novel model, the activation of various cortical, subcortical and brainstem regions and the subsequent release of key neuropeptides play critical roles in the onset of migraine [8]. There are at least three this kind of neuropeptides that have been identified so far, including substance P (SP), calcitonin gene-related peptide (CGRP) and pituitary adenylate cyclase-activating polypeptide (PACAP). Although these neuropeptides share a common property of powerful vasodilation as well as all have been found within the trigeminal afferents innervating the meninges, CGRP is the only one that has been proven as a therapeutic target for the treatment of migraine to date [2, 9, 10]. In this context, the CGRP-targeted therapies, including monoclonal antibodies against CGRP receptor (e.g., Erenumab/AMG 334, Galcanezumab/LY2951742, Fremanezumab/TEV-48125, and Eptinezumab/ALD403), have been either approved or investigated in ongoing phase III clinical trials for the prevention and acute treatment of migraine [11, 12]. The small molecule CGRP receptor antagonists (e.g., olcegepant, atogepant, rimegepant, and ubrogepant) have also been undergoing preclinical or clinical studies [2]. These emerging findings highlight the importance of CGRP not only in a better understanding of the mechanisms underlying the migraine attack but also in the development of medicines specific for migraine.

Upon stimulation by either physical or chemical factors, the trigeminal ganglion neurons (TGNs) produce and release CGRP, which binds to its receptor, a heterodimer complex consisting of the G protein-coupled receptor calcitonin receptor-like receptor (CALCRL) and receptor activity-modifying protein 1 (RAMP1). The binding of CGRP to its receptor complex on cerebrovascular smooth muscle cells, with two cytoplasmic proteins including receptor coupling protein (RCP) and the α-subunit of the G_S_ protein (Gα_S_), activates multiple signaling pathways (e.g., cAMP/PKA and its downstream targets, including KATP channels, ERK, CREB) and results in vasorelaxation and vasodilation [13]. Interestingly, certain glial cells (particularly satellite glial cells around CGRP-expressing neurons) within the trigeminal ganglion also express CGRP receptors, suggesting the existence of a potential neuron-glial communication. While the functional role of this type of intercellular signaling is largely uncertain, it has been found that CGRP can activate the release of nitric oxide (NO), a classic endothelium-dependent vasodilator that is also associated with migraine [14], from ganglion glial cells [15, 16]. Of note, NO can in turn enhance CGRP release, thereby forming a positive feedback loop between CGRP and NO within the ganglion [17]. However, it remains unknown how NO promotes the release of CGRP in TGNs as well as whether this feedback contributes to migraine.

To this end, we sought to investigate the mechanism by which NO regulates the expression and production of CGRP in TGNs. It was observed that exposure to the NO donor S-nitroso-N-acetyl-penicillamine (SNAP) induced the expression and release of CGRP and other migraine-related factors, including substance P (SP), cholecystokinin (CCK), and prostaglandin E2 (PGE2), accompanied by the activation of glycogen synthase kinase 3β (GSK-3β) and the inactivation of PI3K/Akt. These events were markedly attenuated by inhibition of GSK-3β, likely via the inactivation of the NF-κB pathway (reflected by reduced expression and nuclear translocation of RelA/p65). Consistently, NF-κB inhibition also prevented the induction of CGRP by SNAP. Therefore, we identified the GSK-3β signaling pathway as a novel mediator for NO to induce the expression and release of CGRP in TGNs and thus may serve as a potential target for the treatment of migraine.

## RESULTS

### NO induces the expression and production of CGRP in TGNs

**Figure 1:**
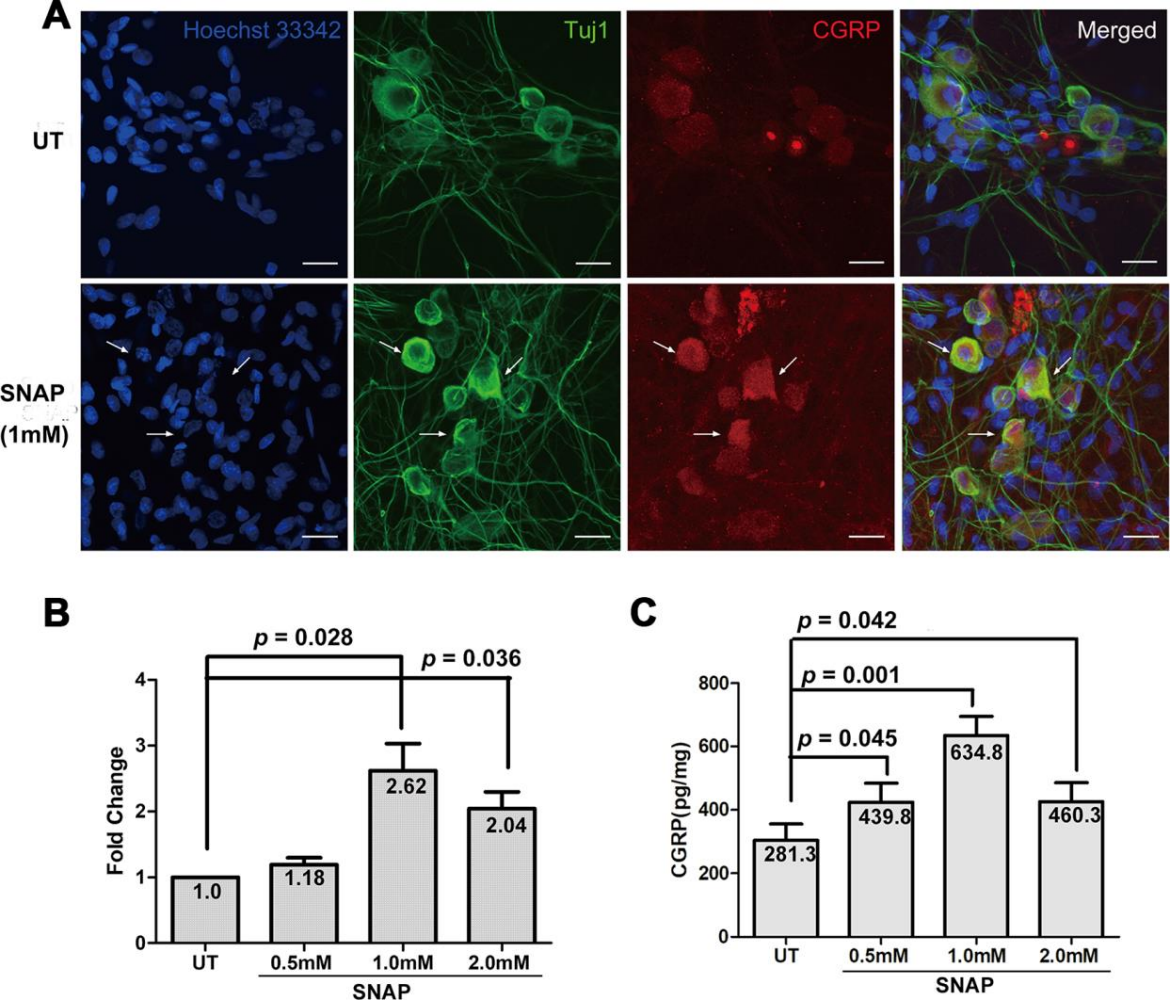
**SNAP induces CGRP expression and production in TGNs.** Primary TGNs isolated from 3-day-old Wistar rats (n = 9) were cultured as described in Materials and Methods and subjected to the experiments as follows. (**A**) Cells were treated with 1.0 mM SNAP for 2 h, followed by culture in NB medium without SNAP for an additional 24 h, after which cells were fixed with 4% paraformaldehyde. Double immunofluorescent staining was then performed using primary antibodies against CGRP (red) and Tuj1 (green, a marker of neurons). Cells were counterstained with Heochst33342 (blue) for nuclei. Scale bar = 20 μm. Arrows indicate representative neurons with increased expression of CGRP. (**B**) Cells were treated with the indicated concentrations of SNAP for 2 h, followed by culture in NB medium without SNAP for an additional 24 h, after which qPCR was performed to monitor the mRNA levels of CGRP. (**C**) In parallel, ELISA was conducted to determine the amount of CGRP protein in culture medium.

It has been reported that while CGRP can activate the release of NO from ganglionic glial cells [15, 16], NO may in turn enhance CGRP release [17]. To validate whether NO could induce the expression and production of CGRP in neurons, the NO donor SNAP was used to mimic NO stimulation in cultured primary TGNs. To do so, TGNs were treated with the indicated concentrations of SNAP for 2 h, and then incubated in the NB medium without SNAP for an additional 24 h. As shown in Figure 1A, immunofluorescent staining revealed that whereas TGNs moderately expressed CGRP primarily in neuronal cell body, exposure to SNAP resulted in a marked increase in TGNs (indicated by arrows). A time-course experiment showed that this effect of SNAP was the most striking when cells were incubated with SNAP for 2 h, compared with those for 1 h or 4 h (Supplementary Figure 1). When immunofluorescent staining for Tuj1 (neuron-specific class III β-tubulin), a marker of neurons, was used to label TGNs, CGRP and Tuj1 were well co-localized in TGNs. In parallel, qPCR was performed to determine the levels of CGRP mRNA in cultured cells. Treatment with ≥ 1.0 mM SNAP significantly induced up-regulation of CGRP expression, compared with that for the control group (Figure 1B; *p* < 0.05 for both 1.0 and 2.0 mM SNAP). The highest level of CGRP transcript was observed after treatment with 1.0 mM SNAP group (ANOVA, followed by Dunnett’s post hoc test, F = 9.217, Q = 4.595; Student’s t test, *p* = 0.028 vs the control group). Alternatively, the protein levels of CGRP in culture medium were also examined by ELISA. After exposed to ≥ 0.5 mM SNAP, a significant increase in the levels of CGRP protein was observed (*p* < 0.05 for all doses), with a peak at 1.0 mM (Figure 1C; F = 5.67, Q = 4.05; *p* = 0.001), consistent with the qPCR results shown in Figure 1B. Together, these results validate the notion that NO stimulation (e.g., by using the NO donor SNAP) could enhance the expression and production of CGRP in TGNs.

### GSK-3β is expressed primarily in TGNs

**Figure 2:**
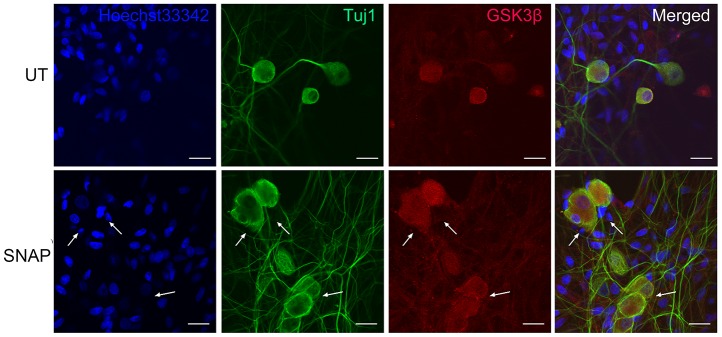
**GSK-3β is predominantly expressed in TGNs.** Cultured primary cells were treated with 1.0 mM SNAP for 2 h, and then cultured in NB medium without SNAP for an additional 24 h. After fixing with 4% paraformaldehyde, double immunofluorescent staining was performed using primary antibodies against GSK-3β (red) and Tuj1 (green), followed by counterstaining with Heochst33342 (blue) for nuclei. Scale bar = 20 μm. Arrows indicate representative Tuj1-negative cells.

GSK-3β, a serine/threonine protein kinase belonging to the glycogen synthase kinase subfamily, participates in the development of the nervous system and the regulation of neuronal functions. Inhibition of GSK-3β has been shown to alleviate neuroinflammation and pain [18], raising a possibility that GSK-3β may also be involved in migraine. Thus, we next examined whether NO-induced expression and production of CGRP in TGNs would be associated with the GSK-3β signaling pathway. To this end, immunofluorescent staining was performed to monitor the expression and localization of GSK-3β in TGNs treated with SNAP. It was observed that GSK-3β was expressed in TGNs with or without SNAP treatment, primarily in Tuj1-positive neurons (Figure 2). However, there were no notable differences in fluorescent intensity for GSK-3β expression between the control and the SNAP treatment groups. Together, these results suggest that NO stimulation does not affect the protein levels of GSK-3β in TGNs.

### NO activates GSK-3β and inactivates AKT

**Figure 3:**
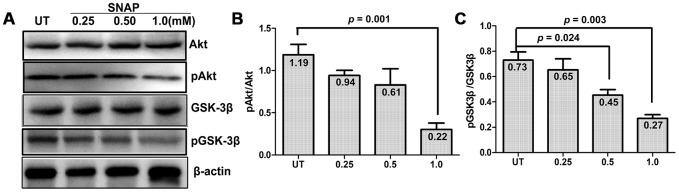
**SNAP induces GSK-3β activation and Akt inactivation.** (**A**) Cultured primary cells were exposed to 0.25 mM, 0.5 mM, and 1.0 mM SNAP for 2 h, followed by incubation in NB medium without SNAP for an additional 24 h, after which cells were harvested and subjected to Western blotting for total and phosphorylated Akt and GSK-3β. (**B**, **C**) Blots were quantified, after which the ratios of phosphorylated vs total Akt and GSK-3β were calculated.

The activity of GSK-3β is mainly regulated through post-translational modifications (mostly phosphorylation) and subcellular localization [18]. GSK-3β can be phosphorylated at multiple residues (e.g., serine 9 and tyrosine 216). While tyrosine 216 phosphorylation increases the activity of GSK-3β, serine 9 (Ser9) phosphorylation is inhibitory and plays a predominant role in the regulation of its kinase activity [19, 20]. Moreover, GSK-3β is a critical downstream target of the PI3K/Akt pathway, in which activated Akt mediates inhibitory phosphorylation of GSK-3β at Ser9 [21]. In this context, Western blotting was performed to examine whether NO stimulation would interfere with this signaling pathway in cultured cells. As shown in Figure 3, exposure to a series of concentrations of SNAP (0.25 - 1.0 mM) barely affected the total protein levels of Akt and GSK-3β. The latter was consistent with the immunofluorescent results for GSK-3β shown in Figure 2. Notably, treatment with 1.0 mM and to a lesser extent 0.25 - 0.5 mM SNAP attenuated both activating phosphorylation of Akt (Ser473) and inhibitory phosphorylation of GSK-3β (Ser9). Quantification of the blots revealed that a significant reduction on the ratios of phosphorylated vs total Akt (Figure 3B) and GSK-3β (Figure 3C) in cells, particularly at the dose of 1.0 mM SNAP (Student’s t test: for Akt, *p* = 0.001; for GSK-3β, *p* = 0.003). Together, these results suggest that NO stimulation could activate GSK-3β in TGNs. They also raise a possibility that this event might be associated with the inactivation of the PI3K/Akt pathway.

### Inhibition of GSK-3β blocks NO-induced expression of CGRP and other migraine-related factors

**Figure 4:**
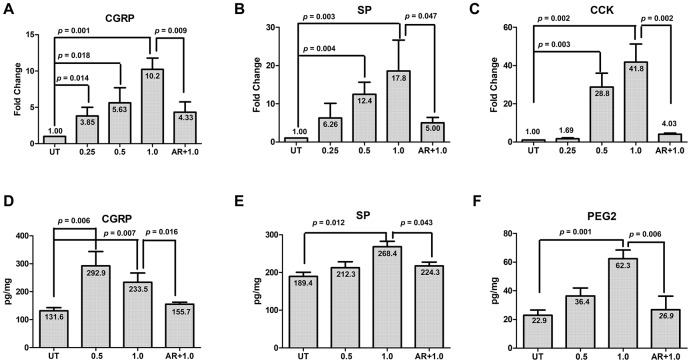
**GSK-3β inhibition abrogates SNAP-induced expression and production of pain-related factors.** (**A**–**C**) Cultured primary cells were exposed to 0.25 mM, 0.5 mM and 1.0 mM SANP for 2 h, or pre-treated with the GSK-3β inhibitor AR-A014418 (10 μM) for 30 min, followed by treatment with 1.0 mM SNAP for 2 h. After culturing in NB medium without SANP, for an additional 24 h qPCR was performed to monitor the expression of CGRP in cells (**A**), SP (**B**) and CCK (**C**). Alternatively, ELISA was conducted to determine the protein levels of CGRP (**D**), SP (**E**) and PGE2 (**F**) in culture medium.

Next, we sought to understand whether activation of GSK-3β functionally contribute to NO-induced expression of CGRP in TGNs. To this end, cultured cells were pre-treated with the GSK-3β inhibitor AR-A014418 (10 μM) for 30 min, followed by incubation with 1.0 mM SNAP for an additional 2 h. After treatment, qPCR and ELISA were performed to monitor the expression and production of CGRP and other migraine-related factors (including SP, CCK and PEG2). In consistence with the results shown in Figure 1, treatment with 0.25 - 1.0 mM SNAP induced the expression of CGRP in cultured cells in a dose-dependent manner (Figure 4A; ANOVA/Dunnett’s test, F = 16.88, Q = 7.94; Student’s t test, *p* =0.001 for 1.0 mM SNAP vs untreated control), accompanied by up-regulation of SP (Figure 4B; F = 6.13, Q = 4.44; *p* = 0.003 for 1.0 mM SNAP vs untreated control) and CCK (Figure 4C; F = 37.15, Q = 9.39, *p* = 0.002 for 1.0 mM SNAP vs untreated control). Of note, pre-treatment with AR-A014418 sharply blocked the expression of CGRP as well as SP and CCK in cells exposed to 1.0 mM SNAP, compared with SNAP alone (Figure 4A–4C; for CGRP, *p* =0.009; for SP, *p* = 0.047; for CCK, *p* = 0.002 for 1.0 mM SNAP with vs without AR-A014418). Furthermore, the protein levels of CGRP as well as other migraine-related factors (SP and PGE2) in culture medium were examined by ELISA. As shown in Figure 4D–4F, while exposure to 0.5 - 1.0 mM SNAP resulted in a marked increase in protein levels of CGRP, SP and PEG2 (for CGRP, F = 16.89, Q = 4.01, *p* = 0.007; for SP, F = 6.208, Q = 4.193, *p* = 0.012; for PGE2, F = 21.95, Q = 7.366, *p* = 0.001 for 1.0 mM SNAP vs untreated control), these events were significantly prevented by pre-treatment with 10 μM AR-A014418 (for CGRP, *p* = 0.016; for SP, *p* = 0.043; for PGE2, *p* = 0.006 for 1.0 mM SNAP with vs with AR-A014418). Together, these results argue that activation of GSK-3β might functionally contribute to NO-induced expression and production of CGRP as well as other migraine-related factors (e.g., SP, CCK and PEG2) in TGNs. They also suggest that GSK-3β might serve as a potential therapeutic target in the treatment of migraine.

### GSK-3β inhibition prevents NO-induced activation of NF-κB in TGNs

**Figure 5:**
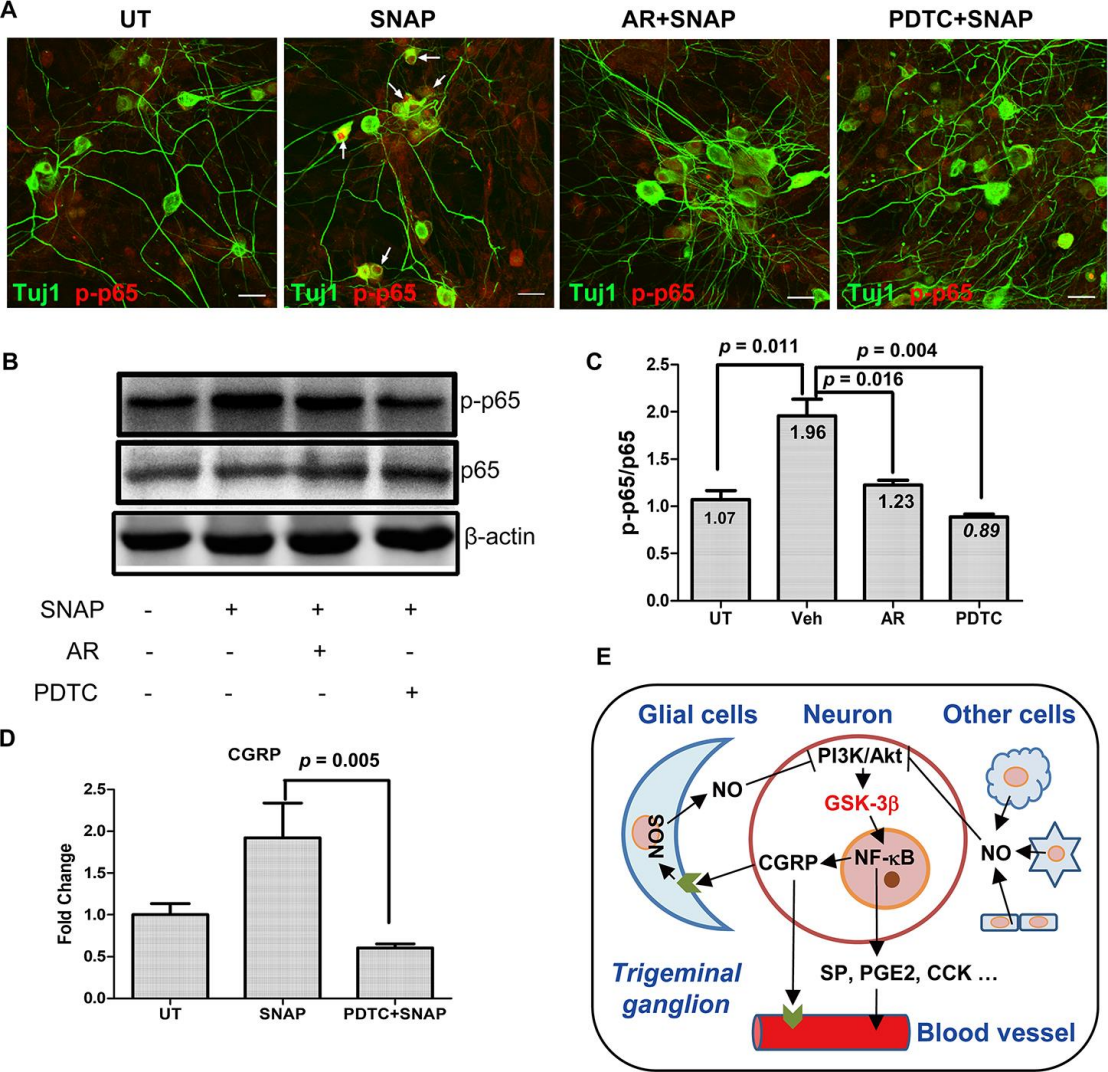
**GSK-3β inhibition blocks NF-κB activation in TGNs exposed to SNAP, preventing SNAP-induced CGRP expression.** (**A**–**C**) Cultured primary cells were pre-treated with 10 μM AR-A014418 or the NF-κB inhibitor PDTC (50 μM) for 30 min, and then exposed to 1.0 mM SNAP for 2 h. After culturing in inhibitor- and SNAP-free medium for an additional 24 h, cells were fixed with 4% paraformaldehyde and double stained with primary antibodies against phosphorylated p65 (red fluorescence) and Tuj1 (green). Scale bar = 20 μm. Arrows indicate representative Tuj1-positive neurons expressing high levels of phosphorylated p65 in the nuclei (**A**). In parallel, Western blotting was performed to monitor total and phosphorylated p65 in cells (**B**). The blots were quantified to calculate the ratio of phosphorylated vs total p65 (**C**). (**D**) In addition, cells were treated with SNAP in the absence or presence of PDTC, after which qPCR was performed to monitor the expression of CGRP in cells. (**E**) A potential model for the mechanism by which NO induces the release of CGPR and other migraine-related factors. Briefly, NO released from glial cells and other types of cells in trigeminal ganglion acts to activate GSK-3β (likely due to inhibition of the PI3K/Akt pathway), which in turn activates NF-κB, thereby inducing the expression and production of CGRP as well as other pain-related factors (e.g., SP, PGE2, CCK, etc.) in TGNs. As a consequence, those factors (particularly CGRP) cause or worsen headache attacks in patients with migraine. Along with previous findings that CGRP released from TGNs may enhance NO production by glial cells, our observations support a positive feedback loop between CGRP and NO in communication between neurons and glial cells.

Whereas migraine is considered as a neurovascular disorder, neuroinflammation also affects the mechanism underlying the pathogenesis of this disease. When stimulated, activated trigeminal sensory neurons can also release pro-inflammatory factors, triggering inflammatory response that leads to peripheral sensitization, a feature of migraine [7]. In this context, we last examine whether NF-κB, a classical signaling pathway mediating inflammation [22], would also be involved in NO-induced activation of TGNs via GSK-3β. As shown in Figure 5A, immunofluorescent staining for phosphorylated p65 (also known as RelA), a core component of the NF-κB complex, revealed that exposure to 1.0 mM SNAP resulted in a marked increase in phosphorylation of p65 in a part of Tuj1-positive TGNs, reflecting the activation of the NF-κB pathway. Strikingly, this event was largely diminished by pre-treatment with the GSK-3β inhibitor AR-A014418 (10 μM) in Tuj1-positive neurons. Analog results were obtained when the NF-κB inhibitor PDTC (50 μM) was employed. Moreover, these phenomena were further confirmed by Western blotting (Figure 5B). Quantification of the blots documented that SNAP increased the ratio of phosphorylated vs total p65 in cultured cells (Student’s t test, *p* = 0.011 for SNAP vs untreated control), while this event was significantly prevented by co-administration of either AR-A014418 (Figure 5C, *p* = 0.016 for AR vs vehicle, *p* = 0.004 for PDTC vs vehicle). Last, inhibition of NF-κB by PDTC also prevented SNAP-induced mRNA expression of CGRP in cells (Figure 5D, *p* = 0.005 for SNAP with vs without PDTC). Similarly, immunofluorescent double staining for phosphorylated p65 and NF200 (neurofilament 200 or neurofilament heavy polypeptide), another specific marker for neurons, revealed that co-administration of PDTC sharply reduced CGRP expression in NF200-positive TGNs (Supplementary Figure 2). Together, these results indicate that NO stimulation might selectively activate the NF-κB pathway in TGNs, which could be prevented by inhibition of GSK-3β, suggesting NF-κB activation as a downstream event of GSK-3β signal during NO-triggered activation of TGNs. They also raise a possibility that GSK-3β-mediated signaling cascade may provide a link between neuroinflammation and migraine.

## DISCUSSION

The neuropeptide CGRP with a potent vasodilating property represents a link between the neurons within the trigeminal ganglion and the meningeal vascular system, which plays a central role in the onset of migraine [2]. While CGRP is abundant in TGNs, it is released from the end of the trigeminal afferents once stimulated. CGRP, as well as other neurotransmitters inflammatory factors such as SP, CCK, PGE2 released from TGNs and other type of cells (e.g., glial cells), leads to the central and peripheral sensitization, thereby triggering the onset of migraine [23]. NO is a well-established universal vasodilator, which is also involved in migraine [24]. While CGRP released from TGNs activates the release of NO from ganglionic glial cells [16], NO can in turn enhance CGRP release from TGNs [25]. However, the mechanism underlying how NO triggers the generation of CGRP in TGNs remains largely unknown. Here we showed that NO stimulation (e.g., by the NO donor SNAP) induced the expression and production of CGRP via activation of GSK-3β in cultured primary TGNs. We also found that blockade of GSK-3β activation reduced CGRP expression and production, at least in part through inactivation of the NF-κB pathway in TGNs. Therefore, these observations raise a possibility that GSK-3β may serve as a potential therapeutic target for the management of migraine.

It is a little known about the relationship between CGRP and NO in migraine. For example, the release of CGRP from peripheral and central nerve endings promotes NO synthesis and trigeminal sensitization, whereas inhibition of inducible nitric oxide synthase (iNOS) can down-regulate CGRP expression and antagonize dilation of meningeal vessels [26, 27]. In rodents, the administration of NO donors can increase the CGRP level, triggering onset of migraine [28, 29]. These findings suggest that a cross-talk between NO and CGRP may facilitate and amplify nociceptive signaling or sensitize neurons to these signals [25]. In this study, we found that the administration of the NO donor SNAP resulted in a marked increase in the expression and production of CGRP *in vitro* in cultured cells, which primarily localized in TGNs. The likelihood that CGRP also distributed in axons of TGNs exposed to SNAP suggests that NO may also facilitate the relay from trigeminal input to second-order neurons of the pain pathway at the central sites, such as spinal trigeminal nucleus in the brainstem and upper cervical levels of the spinal cord, where CGRP-positive axons terminate [30, 31]. The second-order neurons then project to higher cortical pain regions via the brainstem and midbrain [32].

SNAP induces the expression and production of CGRP in a dose-dependent manner, with a peak at 1.0 mM, while this effect was reduced at higher concentrations (e.g., beyond 2.0 mM). SNAP can activate the S-nitrosylation of cysteine residues of the transient receptor potential cation channels TRPA1 and TRPV1 [33], which mediate NO-induced CGRP release and nociception in mice [34]. In this context, high doses of SNAP did not further increase CGRP expression and production because binding and S-nitrosylation of TRPA1 and TRPV1 could be saturated. In addition, excessive NO may result in the generation of peroxynitrite (ONOO-), leading to post-translational modifications (e.g., tyrosine nitration) and inhibition of iNOS expression [35]. This observation may explain why nitroglycerin can aggravate headaches in patients suffering from chronic headaches, while CGRP levels are unassociated with the occurrence of headache [36]. Thus, appropriate concentrations of SNAP or NO may be required to induce the expression and production of CGRP in neurons, as well as to trigger the onset of migraine [37].

To date, the mechanism underlying the interactions between NO and CGRP involving migraine is still poorly understood. As a serine/threonine protein kinase, GSK-3β acts to phosphorylate various substrates, which plays important roles in various cellular processes such as protein synthesis, signal transduction, cell proliferation and differentiation, under physiological and pathological conditions [38–40]. In this study, we observed that GSK-3β was primarily expressed in TGNs. Notably, exposure to SNAP resulted in a marked reduction in inhibitory phosphorylation of GSK-3β at Ser2, indicating its activation. However, treatment with SNAP did not affect the total protein levels of GSK-3β, suggesting its inhibitory phosphorylation plays a predominant role in the regulation of GSK-3β in TGNs after treatment with SNAP. Because the phosphorylation of GSK-3β at the inhibitory site (Ser2) is catalyzed by Akt [20], it is possible that Ser2 dephosphorylation of GSK-3β by SNAP may be associated with Akt inactivation. In this context, exposure to SNAP also inhibited phosphorylation of Akt at an activating site (Ser473) in TGNs, raising a possibility that SNAP-induced GSK-3β activation might be related with the inactivation of the PI3K/Akt pathway. However, there is some evidence suggests that CGRP secretion stimulated by NO does not appear to require the activation of the PI3K signaling pathways, as pre-treatment with the selective PI3K inhibitor wortmannin or LY294002 has no effects on NO-stimulated CGRP secretion by TGNs [24]. Therefore, a possibility that activation of upstream signals other than Akt may also accounts for or contribute to the expression and production of CGRP induced by SNAP in TGNs, via a process dependent upon or independent of GSK-3β, could not be excluded.

It has been reported that inhibition of GSK-3β activity could alleviate pain, probably in association with the inhibition of inflammatory reactions [18]. However, the functional role of GSK-3β in migraine remains virtually unknown. In this study, we found that inhibition of GSK-3β (e.g., by AR-A014418) largely diminished SANP-induced expression and production of CGRP, as well as other known migraine-related factors (e.g., SP, CCK, PEG2) in cultured cells. These observations suggest that activation of GSK-3β signal may be required for NO to facilitate the onset of migraine or increase the severity of headache by promoting expression and production of CGEP and other pain-related factors in TGNs. Therefore, our findings strongly support a notion that GSK-3β may represent a novel target for the prevention and treatment of migraine.

GSK-3β plays a role in the onset and enhancement of neuropathic pain by balancing pro-inflammatory (e.g., TNF-α, IL-1β, and IL-6) and anti-inflammatory factors (e.g., IL-10) via the regulation of cAMP-responsive element-binding protein (CREB) and NF-κB [18, 41]. It has been demonstrated that the protein levels of NF-κB1, CGRP, and TRPV1 are up-regulated in intervertebral disc tissue from patients with degenerative disc disease (DDD) who suffer from chronic back pain. NF-κB is also involved in the mechanisms involving onset or persistence of peripheral nerve pain by regulating the expression of pain-related neuropeptides in DDD patients [42]. In this study, we found that while SNAP induced phosphorylation and nuclear translocation of the NF-κB core component RelA/p65 in TGNs, this event was significantly prevented by pre-treatment with the GSK-3β inhibitor AR-A014418. Moreover, inhibition of the NF-κB pathway (e.g., by PDTC) also prevented the induction of CGRP by SNAP. These results raise a possibility that activation of the NF-κB pathway may represent a downstream event of GSK-3β signal in NO-induced activation of TGNs. However, it could not be excluded that such an action for GSK-3β may also involve other downstream targets.

In summary, SNAP, an exogenous NO donor, could induce the expression and production of CGRP in vitro in cultured primary TGNs, supporting the existence of a positive feedback loop between NO and CGRP, probably involving the communication between neurons and glial cells [25]. The mechanism underlying this event involves GSK-3β activation in TGNs, likely in association with the inactivation of the PI3K/Akt pathway, while inhibition of GSK-3β effectively blocked SNAP-induced expression and production of CGRP and other migraine-related factors (e.g., SP, CCK, and PEG2; Figure 5E). Furthermore, GSK-3β might act to mediate NO-induced activation of TGNs at least in part, via the activation of NF-κB, raising a possible link between neuroinflammation and peripheral or central sensitization during migraine. Together, our observations suggest that GSK-3β may represent a novel target for the treatment of migraine. Therefore, these in vitro findings warrant further validation in vivo in animal models, as well as clinical investigation in patients with migraine, by taking advantage of the availability of various GSK-3β inhibitors currently ongoing clinical trials.

## MATERIALS AND METHODS

### Reagents

The reagents used in this study were purchased from Sigma-Aldrich (St. Louis, MO, USA), including SNAP (a soluble donor of NO [35]), AR-A014418 (a selective GSK-3β inhibitor [41]), and pyrrolidine dithiocarbamate (PDTC; an NF-κB inhibitor [43]). Neurobasal (NB) A medium, Dulbecco’s Modified Eagle Medium/Nutrient Mixture F-12 (DMEM/F-12) and fetal bovine serum (FBS) were obtained from Gibco (Life Technologies, CA, USA). Monoclonal or polyclonal antibodies against NF-κB p65 (Cat# ab16502), phosphorylated p65 (Ser536; Cat# ab86299), GSK-3β (Cat# ab93926), phosphorylated GSK-3β (Ser9; Cat# ab131097), and Tju1 (neuron-specific class III beta-tubulin; Cat# ab78078 and ab18207) were from Abcam (Cambridge, CA, USA). Mouse monoclonal antibody against CGRP (Cat# sc-57053) was purchased from Santa Cruz Biotechnology (Dallas, TX, USA). Protein kinase B (Akt1; Cat# 2938) and phosphorylated Akt1 (Ser473; Cat# #4060) were from Cell Signaling Technology (Beverly, MA, USA). Alexa Fluor 488-conjugated goat anti-rabbit IgG and Alexa Fluor 555-conjugated goat anti-mouse IgG were provided by Invitrogen (Carlsbad, CA, USA). The FastStart Universal SYBR Green Master kit (Cat# 04913850001) and the first-strand cDNA synthesis kit (Cat# 04896866001) were purchased from Roche (Roche Applied Science, Germany).

### Animals

Postnatal 3-day-old Wistar rats were purchased from the Animal Center of Jilin University (certificate number SCXK [JI] 2007-0003). This study was approved by the Ethics Committee of Jilin University, and the animal care and all experimental procedures were in accordance with the institutional guidelines of the Animal Care and Use Committee of Jilin University.

### Culture of primary rat trigeminal sensory neurons

Primary TGNs were obtained from nine Wistar rats. Briefly, the trigeminal ganglia were removed, followed by enzymatic digestion using 2 mg/ml collagenase NB4 (Serva, Germany) and 0.2 mg/ml DNase (Sigma-Aldrich) in DMEM/F12 medium at 37°C for 30-40 min. Cells were collected by centrifugation at 1,000 rpm for 5 min, and plated on poly-l-lysine-coated glass slides in DMEM/F12 medium containing 10% FBS overnight. The slides were then transferred to Neurobasal A medium supplemented with 5% horse serum, 2% B-27 supplement, 0.1 mg/mL of L-glutamine, 100 U/mL penicillin, and 100 L g/mL streptomycin, and incubated for 2 days at 37°C in an atmosphere containing 5% CO_2_. Immunofluorescent double staining for Tuj1 together with GAFP (glial fibrillary acidic protein), a marker of glial cells (e.g., astrocytes), or S100B, a marker of Schwann cells, revealed that a majority of cultured cells were Tuj1-positive neurons, but also contained a small number of S100B-positive cells and to a lesser extent GFAP-positive cells (Supplementary Figure 3).

### Experimental procedures

For each experiment, the isolated TGNs were divided into two groups, including the control and experimental groups. For the former, TGNs were incubated with NB medium without SNAP. For the latter, TGNs were treated with SNAP at a series of concentrations (0.25 mM, 0.5 mM, 1.0 mM, or 2.0 mM) for 2 h and then transferred to the same volume of SNAP-free medium as that in the control group for an additional 24 h. For the experiments involving small molecule inhibitors, cells were pre-treated with or without 10 μM AR-A014418 for 30 min, followed by 1.0 mM SNAP for an additional 2 h. After treatment, cells were harvested and then subjected to enzyme-linked immunosorbent assay (ELISA) to determine the protein levels of CGRP, SP, CCK and PGE2, reflecting the release of these migraine-related factors. In parallel, immunofluorescent staining was performed to examine the localization of CGRP and GSK-3β, and quantitative real-time polymerase chain reaction (qPCR) conducted to determine the mRNA levels of CGRP, SP, CCK and PGE2 in TGNs. Alternatively, cells were lysed and subjected to Western blotting to examine the expression of total and phosphorylated Akt and GSK-3β. In some experiments, 50 μM PDTC was added prior to SNAP treatment, to validate the changes of NF-κB/p65 expression and phosphorylation.

### Quantitative real-time PCR

Total RNA was isolated from TGNs using the Total RNA Miniprep Purification Kit (GeneMark, Taiwan), from which cDNA was prepared using the First-Strand cDNA Synthesis Kit and subjected to qPCR analysis using the FastStart Universal SYBR Green Master Kit. The specific primers used were as follows: CGRP, forward 5′-TCCTGGTTGTCAGCATCTTG-3′ and reverse 5′-CTCAGCCTCCTGTTCCTCCT-3′; SP, forward 5′-GCCCTTTGAGCATCTTCTTC-3′ and reverse 5′-GTCTGAGGAGGTCACCACAT-3′; CCK, forward 5′-TCCGAAGATATGAAGTGCGGC-3′ and reverse 5′-CATCCAGCCCATGTAGTCCC-3′; and glyceraldehyde 3-phosphate dehydrogenase (GAPDH), forward 5′-ATTCCACCCATGGCAAATTC-3′ and reverse 5′-CGCTCCTGGAAGATGGTGAT-3′. The PCR program included an initiation at 95°C for 5 min and then 35 cycles of denaturation at 94°C for 10 s, annealing at 55–59°C for 10 s, and elongation at 72°C for 15 s, followed by elongation at 72°C for 10 min. The specificity of PCR products was verified by melting curve analysis. The mRNA abundance of each target gene was normalized against the house-keeping gene GAPDH for each sample; relative values for mRNA levels were calculated and presented as fold change (2^−ΔΔCT^).

### Immunofluorescent staining

TGNs cultured on cover slips were fixed with 4% paraformaldehyde for 30 min. After washing three times with 0.1 mM phosphate-buffered saline (PBS), cells were permeabilized in 0.1% Triton X-100, blocked in 5% goat serum for 1 h, and incubated with primary antibody (Tuj1, 1:100; CGRP, 1:100; GSK-3β, 1:200; p-p65, 1:100) at 4°C overnight. After washing three times with PBS, cells were incubated with secondary antibody (1:400) for 1 h at room temperature in the dark. Cells were washed three times with PBS, mounted in 50% glycerol. The images were then captured using a laser confocal microscope (Fluo-View FV1000; Olympus, Japan).

### Western blot analysis

TGNs cultured on 6-well plates were lysed with RIPA buffer containing protease and phosphatase inhibitors. After centrifuged, 5× loading buffer was added to the cell lysates and heat-denatured at 90°C for 10 min. 30 μg protein per sample was resolved on 10% sodium dodecyl sulfate-polyacrylamide gels (SDS–PAGE) and then electrically transferred to polyvinylidene fluoride (PVDF) membranes. Membrane was blocked with 5% non-fat milk for 1 h and then incubated with primary antibody (Akt, 1:500; phospho-Akt, 1:1,000; GSK-3β, 1:1,000; phospho-GSK-3β, 1:2,000; NF-κB p65, 1:1,000; phospho-p65, 1:500) at 4°C overnight. After washing three times with tris-buffered saline containing Tween-20 (TBS-T), membranes were incubated with horseradish peroxidase (HRP)-conjugated secondary antibody at room temperature for 1.5 h. Blots were visualized using the Enhanced Chemiluminescence (ECL) reagent (Thermo Fisher Scientific, Waltham, MA, USA). Immunoblots were quantified using the Image J software (U. S. National Institutes of Health, Bethesda, MD, USA); relative protein levels were normalized using actin as an internal control.

### ELISA

The levels of CGRP, PGE2, and SP were examined using the ELISA kits (Elabscience Biotechnology Corp., Wuhan, China), according to the manufacturer’s instructions. Absorbance at 450 nm was detected to determine the levels of these soluble factors. Each sample was measured in triplicate; the concentrations were calculated and expressed as picogram (pg) per gram of total protein.

### Statistical analysis

All statistical analyses were conducted using the GraphPad Prism 5.0 (GraphPad Prism Software Inc, San Diego, CA, USA). Values represent the means ± SEM for at least three independent experiments performed in triplicate. The significance of differences between experimental variables was determined using one-way analysis of variance (ANOVA) followed by Dunnett’s post hoc test. *P* < 0.05 was considered statistically significant.

### Ethics statement

The protocol was approved by the Animal Care and Use Committee of Jilin University.

## Supplementary Material

Supplementary Figures
