# Supplementary material for: NO up-regulates migraine-related CGRP via activation of an Akt/GSK-3β/NF-κB signaling cascade in trigeminal ganglion neurons
Source: Aging (Albany NY). 2020 Apr 10;12(7):6370–84. doi: 10.18632/aging.103031 (PMC7185139; doi:10.18632/aging.103031)
Supplement: Supplementary Figures [file aging-12-103031-s001..pdf]

SUPPLEMENTARY FIGURES

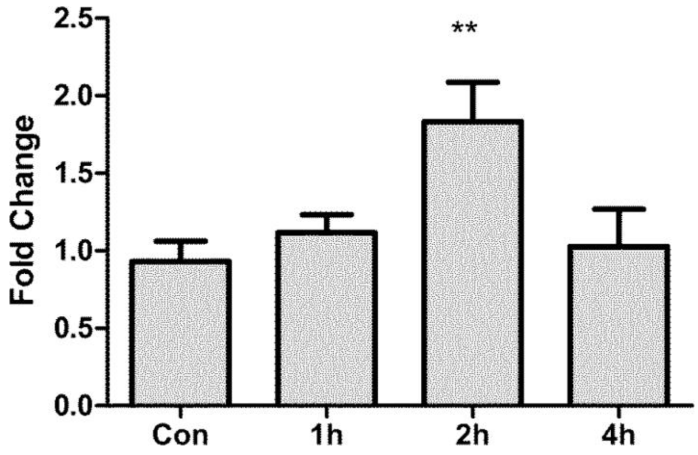

Supplementary Figure 1. SNAP induces CGRP expression in a time-dependent manner. Cultured primary cells were treated with 1.0 mM SNAP for 1 h, 2 h or 4 h, followed by culture in NB medium without SNAP for an additional 24 h. After treatment, qPCR was performed to monitor expression of CGRP in cells. ***p* < 0.01 vs untreated control.

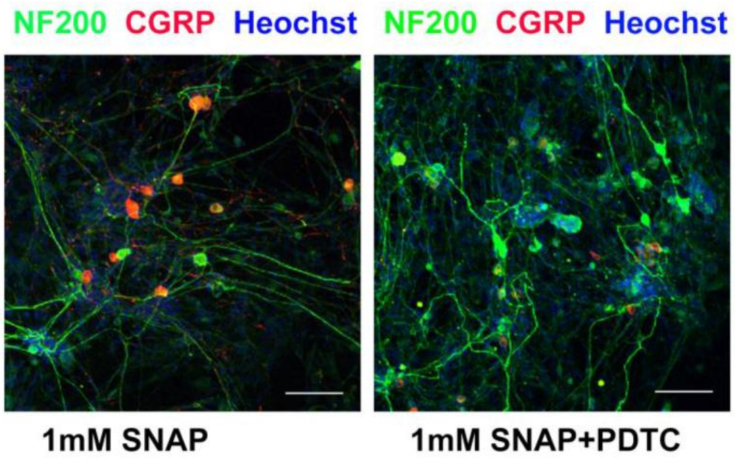

Supplementary Figure 2. The NF-kB inhibitor PDTC prevents SNAP-induced CGRP in TGNs. Cultured primary cells were pre-treated with 50 μM PDTC for 30 min, followed by exposure to 1.0 mM SNAP for 2 h. After culturing in inhibitor- and SNAP-free medium for an additional 24 h, cells were double stained with primary antibodies against CGRP (red fluorescence) and NF200 (green, a marker of neuron) and counterstained with Heochst33342 for nuclei (blue). Scale bar = 20 μm.

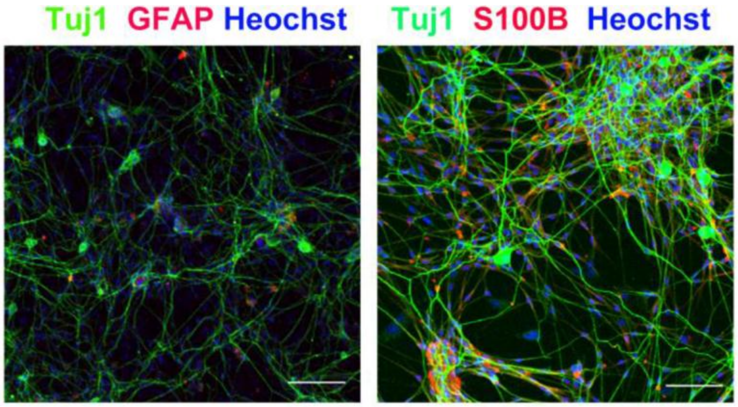

Supplementary Figure 3. Immunofluorescent staining indicates that a majority of cultured cells are neurons in cultured cells. Primary cells obtained from 3-day-old Wistar rats (n = 9) were cultured as described in Materials and Methods, and subjected to double immunofluorescent staining for Tuj1 (a marker of neurons; green) and GFAP (a marker of glial cells; red) or S100B (a marker of Schwann cells; red), and counterstained with Heochst33342 for nuclei (blue). Scale bar = 20 µm.
